# Development of ultrafast single fluorescent-molecule imaging and its application to unravel plasma membrane structure and function in live cells

**DOI:** 10.2142/biophysico.bppb-v23.0018

**Published:** 2026-05-26

**Authors:** Takahiro Fujiwara, Akihiro Kusumi

**Affiliations:** 1 Institute for Integrated Cell-Material Sciences (WPI-iCeMS), Kyoto University Institute for Advanced Study (KUIAS), Kyoto University, Kyoto 606-8501, Japan; 2 Membrane Cooperativity Unit, Okinawa Institute of Science and Technology Graduate University (OIST), Onna-son, Okinawa 904-0495, Japan

**Keywords:** ultrafast single-fluorescent molecule imaging and tracking, ultrafast live-cell PALM and dSTORM, plasma membrane, actin-based membrane skeleton, compartmentalization

## Abstract

Single-molecule imaging in live cells now plays key roles in elucidating molecular dynamics and interactions. However, the imaging time resolution has remained limited, despite its critical importance for precisely capturing molecular events in cells, in contrast with major spatial resolution advances in fluorescence microscopy. To address this issue, we developed an ultrafast camera system that achieves the highest time resolutions reported to date for single fluorescent-molecule imaging and tracking (SFM-IT), reaching fluorophore photophysics-limited (photon-limited) frame times of 33 and 100 μs with single-molecule localization precisions of 34 and 20 nm, respectively, for Cy3, the optimal fluorophore identified. Using this system, we directly detected the hop diffusion of membrane molecules in the plasma membrane, confirming its compartmentalization. Thus, ultrafast SFM-IT has helped to elucidate the principles governing the plasma membrane organization and molecular dynamics. Building on this platform, we further established ultrafast, live-cell, two-color single-molecule localization microscopy (SMLM). This method reduced the data acquisition time by ≈30-fold relative to standard methods, while simultaneously enabling much larger view-fields, with localization precisions of 29 and 19 nm for PALM and dSTORM, respectively. Combining ultrafast SMLM with ultrafast SFM-IT revealed the mesoscopic dynamical organization of focal adhesions (FAs), termed an archipelago architecture, showing that FAs consist of ≈30-nm-diameter FA-protein islands loosely clustered into ≈300-nm-diameter functional units embedded in the compartmentalized fluid membrane (74 nm inside vs. 110 nm outside the FA). Ultrafast SFM-IT and ultrafast SMLM techniques open previously inaccessible spatiotemporal regimes for biophysical cell biology research.

## Significance

The intracellular environment is highly heterogeneous, and even identical molecules behave differently depending on their local context. This is especially evident in the plasma membrane, which contains diverse nano-to-meso-scale functional domains. Many cellular processes arise from transient interactions among a few molecules. These features make single-molecule imaging essential for understanding molecular behaviors in living cells. Yet such events often occur on sub-millisecond to sub-second timescales, far faster than conventional imaging can resolve. This review highlights ultrafast single-molecule techniques achieving ≈30-μs resolution, ≈1,000-fold faster than video rate, with 20–50 nm precision, enabling the direct visualization of molecular dynamics in living cells.

## (A) Development of ultrafast super-resolution single fluorescent-molecule imaging methods in live cells

### Rationale for developing ultrafast single-molecule imaging

Super-resolution fluorescence microscopy based on single-molecule localization, such as photoactivated localization microscopy (PALM), stochastic optical reconstruction microscopy (STORM), and points accumulation for imaging in nanoscale topography (PAINT), has rapidly expanded across biomedical research, including molecular cell biology, biochemistry, biophysics, neuroscience, and immunology [1]. Despite this broad impact, most current single-molecule localization microscopy (SMLM) applications rely on fixed specimens. This is a consequential limitation: unlike electron microscopy, optical microscopy uniquely enables minimally invasive observations of living cells and tissues, and the present SMLM workflow therefore forfeits one of light microscopy’s central advantages. The primary bottleneck is speed. Acquiring a raw single-molecule image sequence, typically on the order of 10,000 frames, to reconstruct a single SMLM image often requires 5–10 min, which is incompatible with many live-cell processes. If SMLM data acquisition could be compressed to shorter than ≈10 s, then super-resolution imaging would become far more broadly applicable to diverse live-cell systems.

In parallel, single fluorescent-molecule imaging and tracking (SFM-IT) in living cells has attracted increasing attention [2]. Historically, its most prominent applications focused on molecules at the plasma membrane, but it is now widely used to interrogate molecular events in the cytoplasm and nucleus, as well as in liquid biomolecular condensates in cells [3–7]. As the scope of SFM-IT has broadened, a recurring constraint has become apparent: many biologically critical events unfold faster than standard imaging can resolve. Numerous critical molecular interactions, including GPCR homo- and hetero-dimerization, are transient, often lasting only on the orders of ≈1–100 ms [8–12]. Moreover, the interactions observed by SFM-IT frequently occur as intermittent, pulse-like episodes, reflecting the repeated short binding events whose frequency and duration distribution, rather than long-lived complexes, dominate the time-integrated cellular output detected by ensemble microscopy. However, to directly resolve the elementary steps of molecular interactions and reactions themselves, and to truly understand the mechanisms at the molecular level, SFM-IT with far higher time resolutions is required. Similarly, non-Brownian motion within condensates can be resolved only when the sampling interval is pushed below the millisecond range [13,14]. In the plasma membrane, the diffusion of phospholipids and transmembrane proteins proceeds as intermittent hops on ≈10-ms time scales [15,16]. Capturing these elementary steps directly, rather than inferring them from time-averaged observables, therefore requires pushing SFM-IT’s temporal resolution into the sub-millisecond regime.

Two broad classes of SFM-IT use distinct localization principles. The first relies on point-spread-function (PSF)-based localization, including single-molecule localization microscopy (SMLM) [1] and other camera-based SFM-IT methods [17–19]. The second class comprises modulation-enhanced localization approaches, including minimal photon fluxes (MINFLUX), structured illumination microscopy-based localization with fluorescence modulation (SIMFLUX), and structured illumination–based point localization estimator (SIMPLE) [20]. These modulation-based methods are conceptually related to the structured-illumination strategies used in stimulated emission depletion (STED) microscopy and structured illumination microscopy (SIM) [21].

Among these, MINFLUX has recently delivered striking biological demonstrations, including 3D localization of protein complexes in mitochondria [22], motor protein stepping in live cells [23], and protein import and export across nuclear pores [24]. *In vitro* or in fixed cells, MINFLUX can achieve ≈1–2 nm localization precision with ≈0.1-ms temporal resolution; for diffusing molecules in the plasma membrane of living cells, the reported localization precision is typically ≈20–50 nm [25,26]. Because MINFLUX localizes molecules by searching for a fluorescence minimum, rather than collecting a full PSF image, its performance can be influenced by background signals (e.g., autofluorescence) and by nearby emitters at high labeling densities; nevertheless, it has also been applied under challenging imaging conditions.

PSF-based localization methods are most often implemented with camera detectors. A principal advantage of camera-based ultrafast SFM-IT is parallelism: unlike MINFLUX, which typically follows one molecule in one color at a time, camera-based SFM-IT can track many molecules simultaneously within a field of view and can readily support multicolor imaging. This capability is crucial when the goal is to capture rare, short-lived encounters and to classify dynamic state switching with adequate statistical power, situations in which throughput and multiplexing can matter as much as nominal localization precision. Thus, the temporal resolution only improves the mechanistic interpretability when sufficient numbers of trajectories can be collected in parallel.

Building on this logic, our group developed an ultrafast camera system capable of detecting and tracking single molecules at temporal resolutions better than 0.1 ms (≥10-kHz frame rate) with a single-molecule localization precision of ≈20 nm [27]. A central goal of this review is to attest that this advance is not merely “faster imaging”; rather, it opens a time domain in which key elementary steps for molecular interactions, rapid diffusion within nanodomains, boundary encounters, and transient intermolecular interactions on microsecond-to-millisecond scales can be directly resolved instead of being inferred from time-averaged observables. In the sections that follow, we show how this ultrafast, camera-based SFM-IT platform (i) reaches the practical, photophysics-limited regime for live-cell tracking, (ii) enables ultrafast SMLM when coupled to PALM/STORM, and (iii) reveals the otherwise inaccessible mechanistic principles of plasma-membrane organization and focal-adhesion dynamics. This review article is an extended version of the Japanese article [28].

### The core technical barrier: Readout noise versus photon-limited signals

Because many mechanistic questions require statistics (state classification, interaction frequencies, dwell-time distributions) and often multicolor co-detection, we must acquire numerous trajectories simultaneously over a usable field of view—not just a single “best” trajectory. This immediately exposes the core constraint in ultrafast SFM-IT: as the frame time shortens, the photons detected per molecule per frame fall rapidly, and single-fluorophore signals approach (or drop below) the root-mean-square (RMS) readout noise generated during pixel charge readout. The challenge is therefore not simply to achieve a high frame rate, but at the same time, to maintain reliable single-fluorophore detectability (and thus localization precision).

Scientific CMOS (sCMOS) cameras achieve low RMS readout noise through rolling-shutter readout and low-noise circuit design, but these choices limit readout speed and constrain practical live-cell frame rates. Industrial CMOS cameras occupy the opposite corner: global-shutter architectures can read all pixels simultaneously at ≥100 kHz, yet their RMS readout noise is tens of times higher than that of sCMOS, making single-fluorophore SFM-IT impractical in their original form. The bottleneck, therefore, was not the frame rate *per se*, but achieving an ultrafast frame rate while preserving a sufficient SNR to localize single fluorophores under photon-starved conditions

**Figure 1:**
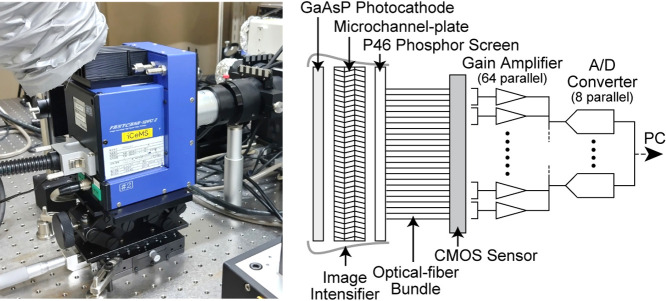
Ultrafast SFM-IT camera system: external view and signal-amplified readout architecture. (A) External view of the ultrafast SFM-IT camera module used for live-cell single-fluorophore detection at ≥10-kHz frame rates. The blue ultrafast camera is attached to a microscope on the right. The gray flexible duct provides cooling air, which is filtered through three HEPA filters. (B) Schematic diagram of the camera readout chain. Photons from single fluorophores are converted to photoelectrons at a GaAsP photocathode in an image intensifier, amplified through a microchannel plate, and reconverted to photons at a P46 phosphor screen. The intensified image is coupled to a high-speed CMOS sensor via an optical fiber bundle, enabling wide-field, global-shutter-style fast readout while keeping single-molecule signals above the effective readout-noise limit. The CMOS output is digitized and transferred to a PC for localization and trajectory analysis.

We overcame this barrier by amplifying the optical signal before it encountered the noisy readout stage [27]. A GaAsP image intensifier was coupled to the CMOS sensor via an optical fiber bundle, and the input (single-fluorophore signal plus background from optics and the live-cell specimen) was amplified at the photocathode until the readout noise became negligible (Figure 1). After photoelectric conversion, the signal was amplified by ≈8,000×, enabling the detection of ≈90% of single-photon-derived photoelectron events in the final image. This key architecture does not make the CMOS sensor intrinsically quieter; rather, it renders the sensor’s readout noise operationally irrelevant.

The architecture shifts the dominant limit. Once the gain is sufficiently high, the performance is set mainly by photocathode quantum efficiency and stochastic gain fluctuations (“excess noise”). The effective GaAsP photocathode quantum efficiency (≈40%) is lower than that of front- and back-illuminated sCMOS (≈70% and ≈90%), and excess noise reduces the localization precision by ≈1.2–1.4× relative to ideal noiseless gain. Accordingly, we embraced a speed-precision trade-off to reach temporal regimes inaccessible to conventional scientific cameras, while maintaining localization precision adequate for nanoscale tracking. Crucially, the limiting resource becomes the number of detected photons per frame. In other words, the platform shifts ultrafast live-cell SFM-IT from a detector-limited regime, where the performance is dominated by faster and lower-noise readout, to a photophysics-limited regime, where the dominant constraint is the fluorophore’s photon yield per frame. This reframing is practical: once the readout noise is no longer a limiting factor, the remaining bottleneck is the number of photons a fluorophore can deliver within each sub-millisecond exposure. In the next section, we quantify this photon budget through dye screening and show that further gains must come primarily from probe chemistry and photophysical kinetics, rather than from faster readout.

### The photophysics ceiling: Dye throughput for ultrafast SFM-IT

**Figure 2:**
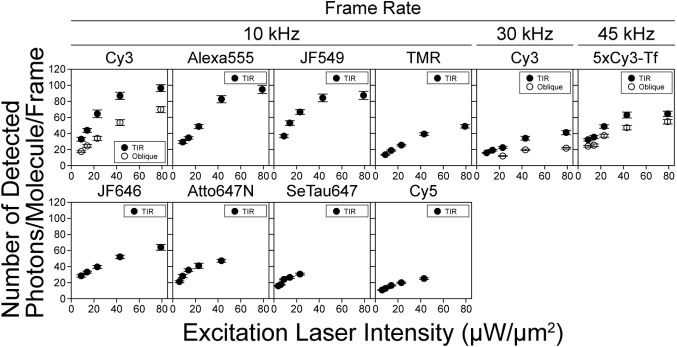
Number of detected photons/molecule/frame as a function of the excitation laser power density for commonly used fluorescent molecules immobilized on glass. ● TIR (total internal reflection) illumination; ○ oblique-angle illumination. Mean±SEM for n=50 spots for each data point. Photon yield increases with excitation intensity but approaches saturation for most dyes, consistent with dark-state (e.g., triplet) entry and recovery kinetics limiting emission on sub-millisecond timescales. Cy3 exhibits the weakest saturation and the highest usable photons/frame in this regime, supporting its selection as an optimal probe for ultrafast SFM-IT, whereas increasing dye numbers per target (5×Cy3-Tf) partially compensates for the photon-per-frame constraint at higher frame rates. Reproduced from [27] and redistributed under CC BY 4.0.

Because the PSF-based localization precision is predominantly determined by the number of detected photons, ultrafast SFM-IT is ultimately governed by photons per frame. We therefore measured photons/molecule/frame for eight dyes commonly used in SFM-IT at frame times of ≤0.1 ms (Figure 2) [27]. Photons/frame increased with excitation intensity only up to a point, and then saturated. The reason is photophysics: entry into the triplet-derived dark state can render a fluorophore non-emissive from microseconds to far longer than seconds via direct and indirect recovery pathways. At high excitation intensities, fluorophores spend a larger fraction of time in these dark states, so additional laser power mainly increases the dark-state occupancy, rather than the photon throughput. Thus, once the detector readout noise is rendered negligible (Figure 1), further performance gains are constrained primarily by fluorophore photophysics. Ultrafast SFM-IT therefore favors fluorophores with high throughput, such as those with short singlet lifetimes and low singlet-to-triplet transition probabilities.

Among the eight dyes tested, Cy3 showed the weakest saturation. For single Cy3 molecules on glass under total internal reflection (TIR) illumination at 79 μW/μm^2^, ≈100 photons were detected within 0.1 ms (10 kHz), yielding ≈20-nm localization precision, whereas ≈40 photons were detected within 0.033 ms (30 kHz), yielding ≈34-nm precision. Operationally, we define “ultrafast SFM-IT” as the SFM-IT at frame rates exceeding 2 kHz (<0.5 ms frame time), which surpass the fastest rates previously achieved for live-cell single-molecule imaging [29], yet still yield ~20–50 nm localization precisions for single fluorophores under practical excitation intensities. Taken together with evaluations across probes and conditions, we concluded that ≈30 kHz represents the practical ceiling for single-fluorophore ultrafast SFM-IT with the current dye photophysics. Although the camera itself can operate at ≥100 kHz, the ceiling is set by the fluorophore photon throughput—i.e., it is a photophysics-limited regime.

Consistent with this, increasing the throughput by labeling transferrin with an average of 5.0 Cy3 dyes (5×Cy3-Tf) yielded ≈60 photons within 0.022 ms (45 kHz) and ≈30-nm localization precision. The implication is straightforward: pushing to higher frame rates will require improved fluorophore throughput and state kinetics, not merely faster readout.

### Ceiling limitations in living cells: Practical photon budgets and usable trajectories

The photophysics ceiling is universal, but living cells determine when it becomes limiting. Excitation intensity cannot be increased arbitrarily without inducing phototoxicity and physiological perturbation, which limits how many photons/frame can be obtained with a given laser power. Cellular autofluorescence, scattering, and non-specific background further reduce the effective SNR, increasing the photon requirement for a given localization precision. Finally, molecular motion penalizes longer exposures: extending the frame time to collect more photons introduces motion blur and trajectory truncation, so the relevant budget is photons per frame, not photons per second.

These constraints directly shape the statistics of usable trajectories. At a 0.1-ms resolution (10 kHz), trajectory-duration statistics under live-cell observation conditions showed that only ≈14% and ≈3% of trajectories exceeded 100 and 300 frames, respectively [27]. This limits the statistical power for diffusion-model tests and for inferring state-switching in live-cell applications. Accordingly, when individual trajectories are short, robustness comes from collecting many trajectories in parallel across the field of view, and, when needed, in multiple colors.

Together, these considerations clarify how “photophysics-limited” becomes “live-cell-limited” operationally: cells restrict excitation due to phototoxicity and elevated background, whereas molecular dynamics require short exposures. The dye photon throughput and state kinetics remain the primary constraints, but the cellular environment defines the operating window over which these limitations can be effectively exploited.

### From instrument development to mechanistic cell biology

This ultrafast, camera-based SFM-IT platform enables wide-field, multicolor-compatible, single-molecule tracking in living cells while operating in a photophysics-limited regime. The central advance is not speed for its own sake, but time-domain access to mechanisms: microsecond-to-millisecond processes, including rapid diffusion within nanodomains, fleeting encounters, and fast switching among dynamic states, can be captured directly rather than inferred. Here, instrument design and cell biology converge: the photon-per-frame constraint, once treated as an engineering limitation, becomes the parameter that defines a mechanistic window into fast molecular dynamics. In the sections below, we first extend the same technical logic to ultrafast SMLM, and then integrate ultrafast SFM-IT and ultrafast SMLM to interrogate the plasma membrane and focal adhesions.

## (B) Ultrafast simultaneous two-color PALM/dSTORM (ultrafast SMLM) in live cells: Compressing minutes of SMLM data acquisition into seconds

### Why SMLM needs speed: The acquisition-time bottleneck and the probe-kinetics limit

Single-molecule localization microscopy (SMLM), including photoactivated localization microscopy (PALM) and direct stochastic optical reconstruction microscopy (dSTORM), reconstructs super-resolution images by accumulating single-molecule localizations over many frames. Its distinctive value is the resulting localization point cloud, which enables quantitative analyses central to cell biology, nanoscale segmentation, cluster/point-pattern analysis, copy-number estimation, and molecular-level colocalization [30]. In many studies, these quantitative readouts are the primary reason for using SMLM.

The major limitation has been the acquisition time. Reconstructing a single SMLM image typically requires single-molecule data acquisition for ≈10,000 frames; at 30 Hz this takes >5 min, which is often incompatible with live-cell dynamics. When the target reorganizes during acquisition, the reconstruction becomes a temporal average that can distort the native architecture. Thus, the bottleneck is not only speed but also interpretability.

**Figure 6:**
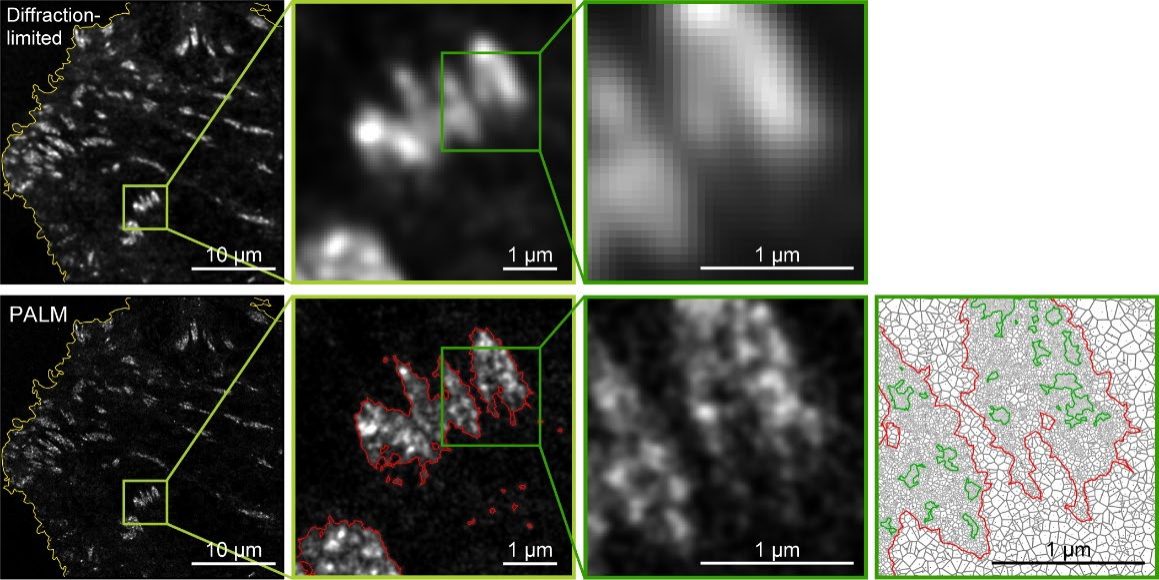
Ultrafast live-cell PALM enables quantitative FA segmentation within seconds. Comparison of diffraction-limited (top) and ultrafast PALM (bottom) imaging of focal adhesions in a live T24 cell expressing paxillin-mEos3.2, obtained in a field of view as large as almost an entire cell (35×35 μm). The PALM reconstruction (acquired at 1 kHz for 10 s) resolves the nanoscale heterogeneity within the FA footprint. The rightmost panel shows the Voronoï-tessellation-based segmentation (SR-Tesseler) defining the FA region (red outline) and the enriched nanoscale subdomains (“islands”; green outlines) used for downstream quantitative analysis. Adapted from [31] and redistributed under CC BY 4.0.

We therefore applied our ultrafast camera system to accelerate PALM/dSTORM by 100–1,000 fold, targeting structures that remain quasi-stationary over ≈10 s (demonstrated in Figures 6 and 7 in later subsections) [31]. However, even with fast hardware, the probe on-off kinetics sets the ceiling: for PALM (mEos3.2) and dSTORM (HMSiR), the on-periods rarely fall below ≈2 ms, making ≈1 kHz (1 ms per frame) the practical limit. Nevertheless, the acquisition duration was reduced from 1–10 min to ≈5–20 s for typical 10,000–20,000 frame data acquisitions at 1 kHz, enabling ultrafast PALM/dSTORM. Operationally, this places live-cell SMLM into a regime where quantitative readouts, including segmentation, clustering, and copy-number inference, can be applied across successive cellular states, rather than a single, minutes-averaged composite.

If probes with much shorter on-periods are developed, then acquisition at ≥10 kHz will be possible and video-rate SMLM (e.g., 30 PALM/dSTORM images per second) will become feasible.

## (C) Hop diffusion in the plasma membrane directly resolved by ultrafast SFM-IT: Actin-based compartmentalization as a universal membrane-dynamics framework

In this section, we show that ultrafast SFM-IT links the compartmentalized architecture of the plasma membrane directly to its universal kinetic consequence, hop diffusion, rather than conflating it into motion-blurred, time-averaged “slow diffusion”.

### Compartmentalized plasma membrane and hop diffusion: What ultrafast tracking established

#### From nanoscale assemblies to a membrane-wide organizing framework

**Figure 3:**
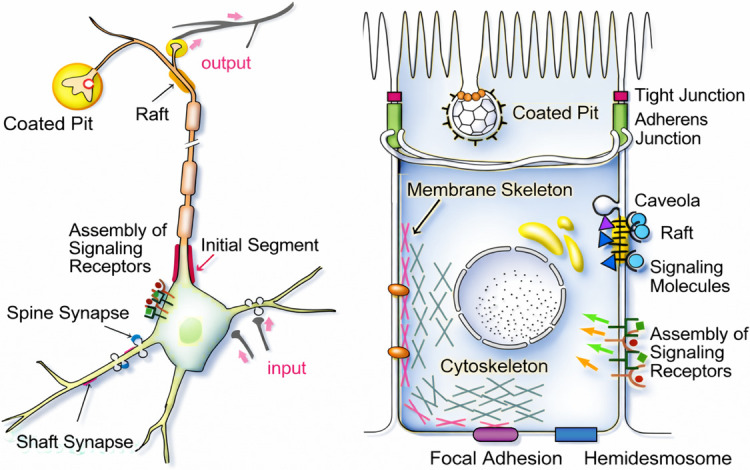
Examples of plasma-membrane domains spanning the mesoscale (3–300 nm) to the micron scale (≥300 nm) in neurons (left) and epithelial cells (right). (Left) Schematic of a neuron highlighting representative membrane domains, including lipid rafts, coated pits, and assemblies of signaling receptors, at synaptic regions (spine/shaft synapses) and the axon initial segment, illustrating the spatial organization of signaling near “input” and “output” sites. (Right) Schematic of a polarized epithelial cell showing the membrane skeleton/cortical cytoskeleton underlying the plasma membrane and major specialized membrane regions—tight junctions, adherens junctions, caveolae/rafts, coated pits, focal adhesions, and hemidesmosomes—where signaling molecules and receptor assemblies concentrate.

Figure 3 illustrates examples of plasma membrane domains ranging from mesoscale (3–300 nm) to micron scale (300 nm–several μm) in neurons (left) and epithelial cells (right). At 3–100 nm scales, transient molecular complexes such as lipid rafts (“Raft”) [32,33] and dimers/oligomers (“Signaling Molecules” and “Assembly of Signaling Receptors”) [34,35] can modulate local diffusion. However, maintaining long-range heterogeneity at spatial scales beyond ≈100 nm requires a membrane-wide organizing framework.

#### How ultrafast gold-particle tracking reveals plasma membrane compartmentalization and hop diffusion

**Figure 4:**
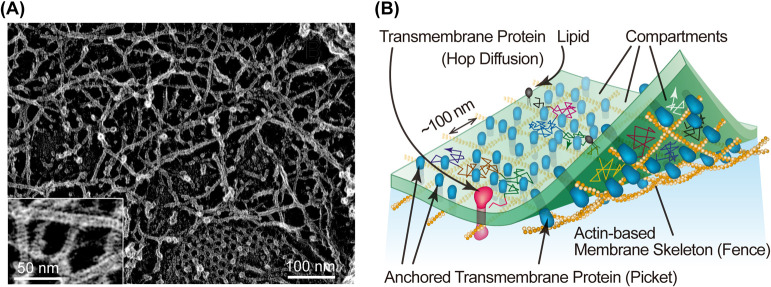
Actin-based plasma-membrane compartmentalization and hop diffusion. (A) Rapid-freeze-deep-etch electron micrograph of the plasma membrane viewed from the cytoplasmic side, revealing a mesh-like “membrane skeleton” closely apposed to the membrane (inset: magnified view of the filament lattice). Adapted from [37] and redistributed under CC BY-NC-SA 3.0. (B) Schematic model in which the actin-based membrane-skeleton “fence” partitions the membrane into ≈100-nm compartments, and rows of transmembrane “pickets” anchored along the fence hinder lateral motion; membrane lipids and proteins diffuse rapidly within a compartment and intermittently cross boundaries by “hop diffusion”. Adapted from [31] and redistributed under CC BY 4.0.

To test this idea experimentally, we performed ultrafast single-particle tracking (SPT) in the early 2000s using 40-nm colloidal gold probes [15,16,36]. Bright-field tracking at 25-μs resolution (40 kHz) revealed short-term confinement within a compartment and intermittent inter-compartmental hops, termed “hop diffusion”, for both phospholipids and transmembrane proteins. The structural basis for this partitioning is visualized in the cytoplasmic-side electron micrograph in Figure 4A, which shows a mesh-like “membrane skeleton” closely apposed to the plasma membrane [37]. The 5.5-nm banding periodicity on individual filaments (inset) is characteristic of actin filaments, indicating that actin is a major component of this cortical scaffold. We found that the short-term confinement arises from the actin-based membrane skeleton (fence), with transmembrane proteins immobilized and aligned along the membrane skeleton (pickets) (Figure 4B).

### Why hop diffusion is expected to be the default mode in a partitioned membrane

In a compartmentalized architecture, hop diffusion is not a special case but the expected consequence of diffusion in the presence of boundaries. A molecule diffuses rapidly within a compartment, frequently encounters the boundary, and only occasionally crosses into a neighboring compartment, by a “hop”. Over long distances, repeated boundary encounters and probabilistic crossing yield an apparently “slow” diffusion, even though motion within each compartment remains fast. Mechanistically, this is explained by the fence-and-picket framework: actin-based “fences” restrict transmembrane proteins via steric collisions with their cytoplasmic domains, while rows of membrane-skeleton-anchored transmembrane “pickets” hinder both proteins and lipids through steric exclusion and increased local hydrodynamic-friction-like effects (Figure 4B).

#### Why ultrafast imaging is indispensable—and why we moved to ultrafast SFM-IT

Temporal resolution is critical because boundary encounters occur on sub-millisecond time scales. A molecule with a microscopic diffusion coefficient of 5 μm^2^/s reaches the boundary of a ≈100-nm-diameter compartment in ≈0.5 ms on average; if the frame time is slower than this, then multiple encounters and hops are averaged into a motion-blurred displacement. Therefore, directly resolving confinement and hops requires sampling at >2 kHz (<0.5 ms per frame), which motivated the development of our ultrafast SFM-IT platform (Figures 1 and 2).

At the same time, gold probes are large relative to target membrane molecules, raising concerns about crosslinking of target molecules on the gold surface and steric hindrance due to collisions with the extracellular matrix. These limitations motivated the development of ultrafast SFM-IT using small fluorescent probes, enabling compartmentalization and hop diffusion to be tested with minimally perturbative labeling and wide-field parallel acquisition under practical live-cell conditions. This sets up the next section, where hop diffusion is directly confirmed from single-fluorophore trajectories in living cells.

#### Biological implications: oligomerization slows hop frequencies, promoting compartmental trapping

Because boundary crossing is probabilistic and sensitive to effective size/drag and to interactions with fences and pickets, oligomerization tends to reduce long-range mobility even when local diffusion inside a compartment remains rapid. When receptors oligomerize in response to signaling, the same architecture can translate a molecular-state transition into a spatial outcome, termed “oligomerization-induced trapping”, measured as enhanced confinement and longer residency within particular compartments. Fully developing these biological consequences would require a dedicated review; here, we only note oligomerization-induced trapping as an illustrative example to clarify why directly observing hop diffusion matters.

### Ultrafast SFM-IT confirms hop diffusion using small fluorescent probes

**Figure 5:**
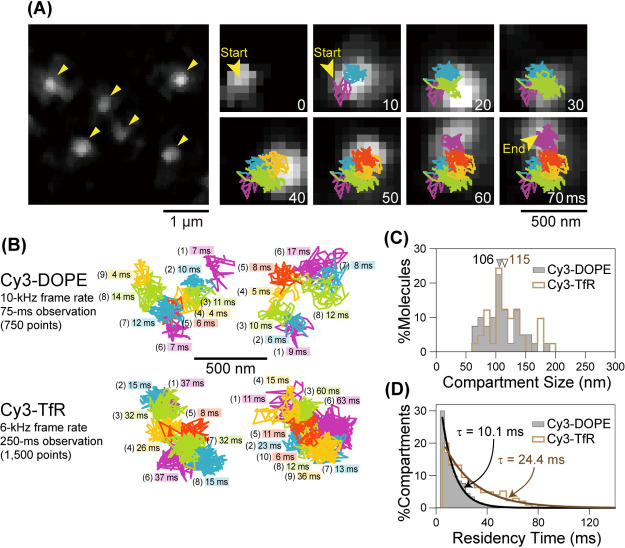
Hop diffusion directly resolved by ultrafast SFM-IT in the apical plasma membrane. (A) (Left) A typical snapshot of single Cy3-DOPE molecules (arrowheads) in the apical plasma membrane of T24 cells visualized by the oblique-angle illumination and ultrafast SFM-IT with a 0.1-ms frame time. (Right) Representative time series (every 10 ms) of a Cy3-DOPE molecule tracked at a 0.1-ms resolution; the trajectory segment within each membrane compartment is color-coded to visualize successive confinement and hopping events. (B) Representative trajectories illustrating hop diffusions of Cy3-DOPE and Cy3-transferrin receptor (Cy3-TfR). Numbers in parentheses indicate the order of compartments visited, and the values denote the residency time spent in each compartment in milliseconds. Panels (C) and (D) show the distributions of the compartment sizes (arrowheads indicate the medians) and the residency times within each compartment, respectively, as quantitatively determined for Cy3-DOPE and Cy3-TfR based on a hop-diffusion model. Adapted from [27] and redistributed under CC BY 4.0.

Using the developed camera system described in Figure 1, we performed ultrafast SFM-IT on the apical plasma membrane of human epithelial T24 cells using the oblique-angle illumination [27]. We tracked (i) a phospholipid analog, Cy3-DOPE, and (ii) a transmembrane protein, transferrin receptor labeled with Cy3-transferrin (Cy3-TfR). The trajectories display a characteristic pattern: rapid diffusion within a ≈100-nm domain, occasional escape, and re-confinement in an adjacent domain—i.e., hop diffusion was observed directly (Figure 5A, B). This trajectory phenotype is the experimental counterpart of the conceptual scheme in Figure 4B.

We quantified this behavior using MSD–Δt (mean squared displacement as a function of time) analysis and by fitting with a hop-diffusion model [38]. For Cy3-DOPE, we employed an observation frame rate of 10 kHz and found that trajectories on the order of 1,000 frames were required to reduce the statistical error in the MSD values and achieve accurate fitting. Because the hop rate of TfR was expected to be slower than that of Cy3-DOPE, we reduced the frame rate to 6 kHz for TfR and lowered the excitation intensity accordingly. This enabled us to obtain trajectories on the order of 1,000 frames with localization precision similar to that for Cy3-DOPE, and to perform the hop-diffusion analysis with comparable accuracy. The median compartment sizes were similar (≈110 nm) for DOPE and TfR (Figure 5C), indicating that both probes experience the same underlying compartment geometry expected from the actin-based partitioning model. However, the residency time constants within one compartment differed: ≈10 ms for DOPE vs. ≈24 ms for TfR (Figure 5D). The combination of a similar compartment size with a ≈2.4-fold longer residency time for TfR is also consistent with the fence-and-picket mechanism: actin-based “fences” primarily constrain transmembrane proteins, while rows of membrane-skeleton-anchored “pickets” hinder both proteins and lipids (Figure 4B). Thus, ultrafast SFM-IT provides a minimally perturbative, fluorescence-based confirmation of hop diffusion in the same mechanistic language as Figure 4B, but now read out directly from single-fluorophore trajectories as in Figure 5B. Hop-diffusion parameters did not differ significantly between apical and basal membranes.

### Convergence and distinction relative to MINFLUX

A recent MINFLUX application for tracking membrane molecules, optimized for ≈0.6 ms temporal resolution with ≈7 nm localization precision, reported confined trajectories consistent with hop diffusion in 50–100 nm domains after deep-learning-assisted analysis [39], converging conceptually with the compartment framework (Figure 4B) and the hop-diffusion signatures observed by ultrafast SFM-IT (Figure 5B). At the same time, the two approaches remain practically complementary. Camera-based ultrafast SFM-IT provides wide-field parallelism and multicolor readiness, allowing many trajectories to be collected simultaneously (as in Figure 5A) and thereby enabling robust population statistics and interaction-ready experiments (as in Figure 5B, C, D). MINFLUX, in contrast, generally enables much longer trajectories, such as 1,000–2,000 steps on average at ≈0.6-ms time resolution, which is advantageous for resolving extended state sequences within individual trajectories [39].

## (D) A multi-scale “archipelago” architecture in focal adhesions: ultrafast SMLM maps and ultrafast SFM-IT trajectories

Ultrafast SMLM defines the nanoscale architecture, whereas ultrafast SFM-IT reveals how molecules traverse that architecture and are transiently captured. Together, these approaches bridge the apparently static super-resolution structure with live, rate-limiting molecular exchange.

### Ultrafast SMLM reveals live-cell FA nano-architecture and enables quantitative segmentation

Focal adhesions (FAs) are micron-scale plasma-membrane domains that mediate cell–extracellular matrix adhesion and are responsible for cell migration (“Focal Adhesion” in Figure 3). As shown in the SMLM image in Figure 6, an FA “region” is first operationally defined as the membrane area enriched in FA proteins; within that region, we consistently observe smaller nanoscale sub-FA domains where specific components are further concentrated. For clarity, we refer to these nanoscale sub-FA domains as FA-protein “islands”. Ultrafast SMLM makes these nested organizational levels quantifiable and comparable across proteins and conditions [31].

#### Nanoscale segmentation in live cells (ultrafast PALM)

Figure 6 shows an ultrafast PALM image of FAs in the basal membrane of a live T24 cell, obtained by the TIR illumination and data acquisition at 1 kHz for 10,000 frames (10 s) using paxillin fused to mEos3.2. Using a Voronoï-tessellation-based cluster analysis (SR-Tesseler) [40], the FA region was identified as a paxillin-enriched area (red contours), and within that FA region, more strongly paxillin-enriched nanoscale sub-FA domains were segmented as “islands” (green contours). After accounting for the 29-nm localization precision of mEos3.2, the characteristic island diameter was estimated to be ≈30 nm [31]. This ≈30-nm length scale is not merely descriptive: it provides a transferable metric for comparing different FA proteins, perturbations, and FA subregions on a common scale.

#### Estimating molecule numbers per island: Turning a shape into a quantitative unit

To estimate the paxillin copy number per island in wild-type (non-transfected) cells, we evaluated (i) the expression ratio (transfected vs. endogenous), (ii) the detection efficiency (photoconversion efficiency for PALM; labeling efficiency for dSTORM), and (iii) the repeated detections due to blinking. Independent estimates from PALM (mEos3.2-paxillin) and dSTORM (HMSiR-labeled paxillin) converged on ≈30 paxillin molecules per island [31]. This agreement argues against probe- or pipeline-specific artifacts and anchors the “island” concept not only morphologically but also quantitatively.

### Ultrafast, simultaneous two-color SMLM: Mapping multiple FA components and hierarchical clustering

**Figure 7:**
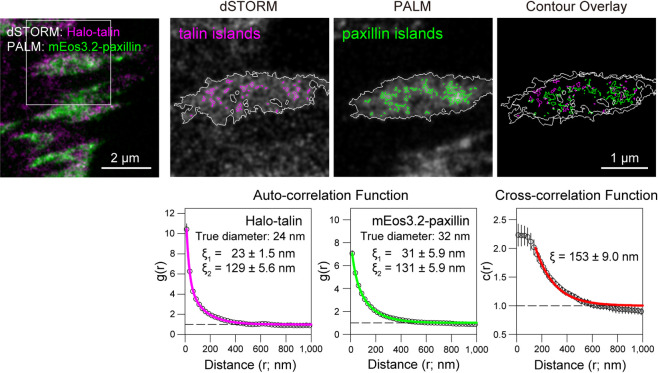
Two-color ultrafast dSTORM/PALM reveals nanoscale FA “islands” and their mesoscale clustering. Two-color SMLM of focal adhesions using dSTORM of HMSiR-labeled Halo-talin and PALM of mEos3.2-paxillin (top; examples and contour overlay). Localizations were analyzed by auto-correlation (talin; paxillin) and cross-correlation (talin vs. paxillin) functions (bottom). Island size was estimated from twice the exponential correlation length, with correction for localization precision (19 nm for dSTORM and 29 nm for PALM). Data are shown as mean±SEM (n=25 FAs for each data point). The correlation analyses support a model in which ≈30-nm-diameter talin and paxillin islands are loosely clustered within ≈300-nm regions. Adapted from [31] and redistributed under CC BY 4.0.

To extend island/cluster mapping beyond a single marker, we developed an ultrafast, simultaneous two-color SMLM workflow that pairs the ultrafast PALM of paxillin-mEos3.2 with the ultrafast dSTORM of HMSiR-labeled FA proteins in the same field of view [31]. Using this approach in mouse embryonic fibroblasts, Figure 7 shows a representative dataset combining talin dSTORM with paxillin PALM. Talin formed ≈30-nm islands that partially, but not extensively, overlapped paxillin islands. Spatial autocorrelation revealed two correlation lengths: a shorter one corresponding to the ≈30-nm islands (after accounting for the localization precision) and a longer one consistent with loose clustering into ≈300-nm units. Thus, the FA organization naturally separates into two coupled length scales: ≈30 nm islands and ≈300 nm loose clusters.

Spatial cross-correlation between talin and paxillin indicated overlap at the level of loose clusters, suggesting co-enrichment within shared ≈300-nm subregions even when the ≈30-nm island-level overlap is limited. Similar results were obtained when dSTORM targeted FAK or vinculin instead of talin. A parsimonious interpretation is that the limited copy numbers per ≈30-nm island reduce the detectability of island-scale overlap, whereas the mesoscale co-organization remains robust, which is precisely the distinction that simultaneous two-color mapping can resolve.

Long-term dSTORM for 60 seconds further showed that paxillin recruitment is synchronous among islands within a loose cluster but not across clusters, implying that the ≈300-nm loose cluster can behave as a functional recruitment unit for paxillin (and possibly other cytoplasmic components) [31]. Methodologically, this is a key payoff of ultrafast SMLM: by acquiring super-resolution localizations in live cells rapidly enough to revisit the same FA neighborhood over tens of seconds, one can extract not only the spatial “archipelago” map but also the local, near-neighbor recruitment dynamics within that map. Here, faster acquisition does not merely shorten imaging time; it reveals how constituent molecules are recruited and released at single-molecule resolution.

### Ultrafast SFM-IT resolves inter-island molecular dynamics and reveals finer compartmentalization inside FAs

**Figure 8:**
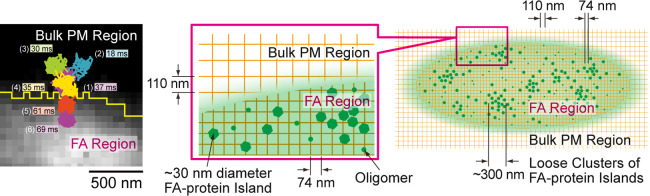
Hop diffusion of transmembrane proteins persists inside focal adhesions and supports a model of an “archipelago of FA-protein islands in the compartmentalized fluid”. (Left) Representative trajectory of TMR-labeled Halo-TfR tracked at a 0.167-ms resolution as it enters the FA region (marked by mGFP-paxillin) from the surrounding bulk plasma membrane (PM). (Right) Schematic model of the FA archipelago architecture: FA-protein islands of various molecular stoichiometries with a diameter of ≈30 nm form loose clusters with a diameter of ≈300 nm in the fluid membrane, which is partitioned into 74-nm compartments (110 nm in the bulk PM). These compartment boundaries are probably composed of the actin-based membrane-skeleton mesh (schematically shown by the orange lattice), which might be bound and stabilized by various FA proteins as monomers, oligomers, and islands. Adapted from [31] and redistributed under CC BY 4.0.

We next investigated the inter-island “channels” within FAs, which are predominantly composed of fluid membrane, by tracking a non-FA transmembrane protein, TfR, in the basal membrane of T24 cells using the TIR illumination and ultrafast SFM-IT at 0.167-ms resolution (6 kHz) (Figure 8, left) [31]. TfR molecules initially located in the bulk plasma membrane continued exhibiting hop diffusion upon entering the FA region. Hop-diffusion analysis of MSD-∆t for individual trajectories revealed that the median compartment size decreased from 110 nm in the bulk membrane to 74 nm inside FAs, demonstrating that the inter-island fluid membrane within FAs is itself compartmentalized (Figure 8, right). The residency time constant within a compartment was prolonged from 24 ms in the bulk membrane to 36 ms inside FAs, consistent with the idea that FA proteins and their oligomers densely bind to the finer actin-based meshwork, thereby stabilizing the meshwork and/or sterically hindering boundary crossing (Figure 8, right). Integrins, the major adhesion receptors in FAs, likewise exhibited hop diffusion inside FAs and, in addition, frequently displayed transient immobilizations on FA-protein islands, with dwell lifetimes ranging from milliseconds to tens of seconds [31,41]. The archipelago architecture of the FA-protein islands, based on the compartmentalized fluid membrane, is considered biologically significant because it would allow for integrin’s rapid recruitment to and removal from the FA-protein islands, thereby facilitating the dynamic regulation of FA formation, reorganization, and disintegration [42–44].

### Integrating maps and trajectories: How an FA can be structured yet rapidly exchangeable

Taken together, the integration of ultrafast SMLM and ultrafast SFM-IT defines a unified, testable picture: a micron-scale adhesion domain is built from nanoscale protein islands (≈30 nm) packed into mesoscale units (≈300 nm), embedded in, and coupled to, a dynamically partitioned fluid membrane. This combination resolves an apparent paradox that has long complicated intuitive models of FAs: how a micron-scale domain can maintain robust spatial enrichment while still permitting rapid molecular exchange. In this framework, ultrafast SMLM supplies the quantitative “where”, and ultrafast SFM-IT supplies the kinetic “how”: the inter-island membrane acts as a structured traffic field, rather than a featureless solvent.

In particular, ultrafast SFM-IT shows that molecules traverse the inter-island membrane by hop diffusion even within the FA region, but with finer compartmentalization and longer residency times than in the surrounding membrane. This provides the missing kinetic counterpart to the apparently static SMLM-defined island/cluster map: nanoscale enrichment is embedded in, and dynamically coupled to, a partitioned membrane that regulates traffic and capture.

## (E) Why diffusion suppression and domain hierarchy matter

### From the fluid–mosaic model to heterogeneity

Singer and Nicolson’s fluid–mosaic model, published in 1972, established the foundational idea that the plasma membrane is a two-dimensional fluid in which lipids and proteins diffuse laterally [45]. This shift, from static composition to lateral dynamics, set the stage for a central question in membrane biology: how a “fluid” membrane nonetheless produces reproducible spatial organization and function. As experimental methods improved, it became clear that living-cell membranes are not featureless liquids. Instead, they exhibit persistent heterogeneity spanning nano- to meso-scales (Figure 3), and many key cellular outcomes depend on where and when molecules meet, cluster, and exchange.

### Actin-based compartmentalization as a unifying framework

Many factors contribute to membrane heterogeneity, including transient nanodomains such as lipid rafts (“Raft” in Figure 3), reversible dimerization/oligomerization (“Signaling Molecules” and “Assembly of Signaling Receptors” in Figure 3), and interactions with extracellular structures. However, to account for the robust suppression of long-range diffusion (>30 nm) across the entire cell surface, the most general and dominant framework is compartmentalization by the actin-based membrane skeleton (Figure 4). The cytoplasmic-side ultrastructure provides the physical basis: a mesh-like cortical scaffold closely apposed to the plasma membrane, largely composed of actin filaments as indicated by the characteristic 5.5-nm banding periodicity (Figure 4A). In this view, “hop diffusion” (Figure 4B) is not a special case of membrane diffusion but the generic dynamical signature of a partitioned membrane. Because many downstream phenomena, such as apparently slower diffusion relative to diffusion in artificial membranes, immobilized fractions, and state-dependent motility shifts, are interpreted through this framework, readers need an accurate, quantitative understanding of compartmentalization for assessing mechanisms, rather than relying on qualitative intuition.

### Why sub-millisecond tracking is required

Over time, the membrane field methodologically progressed by learning to measure not only who is near whom, but also how molecules move and exchange. Interaction/proximity assays (e.g., DRM fractionation, FRET/BRET, proximity ligation assay, and proximity labeling [46–49]) report neighborhood- or distance-dependent signals with distinct operational meanings; as such they are powerful but do not directly resolve the elementary dynamic steps that generate heterogeneity. For dynamics, ensemble approaches such as FRAP, FCS, and their extensions (spot-variation FCS, STED-FCS, image correlation spectroscopy [50–52]) infer mobility from intensity changes or fluctuations in a fixed observation volume, whereas single-molecule tracking yields trajectories that expose heterogeneity, mode switching, and rare events at the level of individual molecules. Crucially, the compartment framework imposes a hard temporal requirement: a molecule diffusing at a diffusion coefficient of 5 μm^2^/s reaches the boundary of a 100-nm-diameter compartment in ≈0.5 ms on average. If sampling is slower than this, then boundary encounters and hops collapse into motion blur and can be misread as time-averaged “slow diffusion”. This is precisely why sub-millisecond tracking is not a luxury, but the minimum requirement for directly testing compartmentalization and hop diffusion in living cells, and why this experimental demand emerged from membrane biology itself.

### A unified workflow: Maps plus trajectories

Against this historical and conceptual background, the platform reviewed here is best understood as a practical as a practical “maps+trajectories” workflow. By making the photons-per-frame constraint explicit, it enables camera-based SFM-IT at frame rates exceeding 2 kHz, the minimum required to directly resolve compartment-and-hop dynamics (Figures 1, 2, and 5) [27]. In parallel, ultrafast live-cell SMLM compresses nanoscale mapping into acquisition windows short enough that super-resolution “maps” can be captured as distinct cellular states rather than minutes-averaged composites (Figures 6 and 7) [31]. Used together, these two modes link nanoscale architecture to exchange and mobility within the same functional domain (Figure 8), turning membrane heterogeneity from a qualitative descriptor into a quantitatively testable mechanism.

In practice, the choice and integration of single-molecule tracking and super-resolution imaging methods should be guided by the spatiotemporal scale of the event of interest, the precision required to resolve it, and the statistics needed for robust inference. Table 1 summarizes typical performance metrics of currently available SFM-IT and SFM-IT-based super-resolution imaging methods, including MINFLUX, in live-cell applications. The comparison highlights why ultrafast SFM-IT combined with ultrafast SMLM is often a particularly practical approach for live-cell studies: ultrafast SFM-IT provides high-parallelism trajectories that capture rapid motion, encounters, and state transitions across a wide field of view, whereas ultrafast SMLM enables nanoscale maps within acquisition time windows compatible with evolving cellular states. Used together, these approaches can relate “how molecules move” to “where molecules are organized” over experimentally compatible spatiotemporal ranges.

MINFLUX provides an additional, complementary route. Under suitable conditions, MINFLUX tracking can offer higher localization precision and longer trajectories compared with camera-based SFM-IT, whereas MINFLUX imaging can reach molecular-scale spatial resolution in the single-digit-nanometer range, approaching regimes that are challenging for SMLM and thus aligning naturally with structural questions. The recent demonstration of tracking protein import and export across nuclear pores [24] represents a landmark achievement that takes advantage of these strengths. At the same time, the lower parallelism and longer acquisition times typically associated with MINFLUX imaging can make it less straightforward to combine with tracking in the same live-cell workflow [26]. Overall, Table 1 clarifies that camera-based ultrafast methods and MINFLUX occupy complementary regions of the experimental design space—one emphasizing wide-field, multicolor, high-throughput trajectories and rapid map acquisition, and the other emphasizing long trajectories and molecular-scale mapping when background and labeling density permit.

## Conclusion

Taken together, the developments surveyed here shift the center of gravity of single-molecule cell biology from static description to time-resolved mechanism. When trajectories are sampled fast enough to preserve elementary steps, boundary encounters, brief binding, and rapid switching, diffusion is no longer a nuisance to be averaged out, but a readable signature of underlying organization. Ultrafast SFM-IT brings this regime within reach by making the photon budget explicit and by operating at the photophysics ceiling, where further progress is dictated by probe kinetics, rather than detector electronics. Ultrafast live-cell SMLM, in turn, compresses nanoscale mapping into acquisition windows short enough that “structure” can be treated as a transient cellular state. Their integration closes a key conceptual loop: nanoscale architecture and sub-millisecond dynamics can be measured in the same living system and interpreted within a shared quantitative framework, from the plasma membrane compartmentalization to the mesoscale organization of focal adhesions. The broader message is practical: once photons per frame are accounted for explicitly, the experimental design space becomes transparent, and previously inaccessible spatiotemporal regimes become routine targets for hypothesis-driven cell biophysics.

## Conflict of interest

The authors declare no conflict of interest.

## Author contributions

Takahiro Fujiwara: writing—original draft. Akihiro Kusumi: writing—review & editing.

## Data availability

The evidence data generated and/or analyzed during the current study are available from the corresponding author on reasonable request.

## Figures and Tables

**Table 1:** Summary of “typical” performance metrics of available SFM-IT and super-resolution (SR) imaging methods in their application to live-cell studies

Method	Live-cell compatibility	Parallelism (multiple-molecule/multicolor)	Field of view (approximate diameter)	SFM localization precision	SFM temporal resolution	SFM tracking duration	SR image spatial resolution	Single SR image acquisition time	References
SFM-IT	high	high	50 μm	10–30 nm	1–100 ms	100 frames	—	—	27
ultrafast SFM-IT	high	high	30 μm	30–50 nm	0.1–1 ms	300 frames	—	—	27
MINFLUX tracking	high	low	5 μm	7–50 nm	0.1–0.6 ms	1,000–2,000 frames	—	—	25, 39
SMLM	low	high	50 μm	5–20 nm	10–100 ms	—	12–50 nm	minutes–hours	1
ultrafast SMLM	middle	high	30 μm	20–30 nm	1–4 ms	—	50–70 nm	0.5–10 s	31
MINFLUX imaging	low	low	5 μm	1–2 nm	0.1–1 ms	—	single-digit nm	minutes–tens of minutes	26
